# Postpartum intrauterine device placement: a patient-friendly option

**DOI:** 10.1186/s40834-018-0057-x

**Published:** 2018-04-15

**Authors:** Carrie Cwiak, Sarah Cordes

**Affiliations:** Division of Family Planning, Department of Gynecology and Obstetrics, 49 Jesse Hill Jr. Drive SE, Atlanta, GA 30303 USA

**Keywords:** Intrauterine device, Postpartum, Contraception, Family planning

## Abstract

Women in the United States are increasingly choosing an intrauterine device (IUD) for contraception. Since the postpartum period is an important time to consider a patient’s need for contraception, offering postpartum IUD placement is considered best practice. Effective implementation of postpartum IUD placement occurs within a context of shared decision making wherein patients are given full information about all options and guided to methods that best fit their lifestyle. Within this context, both the non-hormonal and hormonal IUDs are safe, highly effective, well tolerated, and convenient options. National guidelines support the placement of IUDs, whether immediate (within 10 min of placental delivery) or early postpartum (after 10 min and before 4 weeks after placental delivery), for breastfeeding or non-breastfeeding women. Studies have noted increased IUD expulsion rates, but equivalent IUD usage rates with immediate or early postpartum placement. Postpartum placement requires additional skills that can be easily taught. Finally, successful implementation of a postpartum IUD placement program can be accomplished in hospitals using a team-based approach.

## Background

In the United States (US), 45% of all pregnancies are unintended. Those most at risk include women with: low socioeconomic status, low education level, minority status, cohabitating status, and younger age [1]. The US unintended pregnancy rate has been recently dropping after staying stagnant for decades, in part due to the increased use of effective contraception among adolescents and adults. The biggest increase was observed in the use of long acting reversible contraception (LARC), which includes intrauterine devices (IUDs) and implants, noted among both multiparous and nulliparous women. The biggest decrease was seen in the use of sterilization. In particular, the US National Survey of Family Growth noted an increase in IUD use from 5.6% in 2008 to 11.8% in 2014, making IUDs now the fourth most common contraceptive method used in the US [2].

## Postpartum IUD Placement

The postpartum period is an important time to consider a patient’s need for contraception. Over 21 countries, nearly two thirds of patients report an unmet need for family planning within 2 years postpartum [3]. The mean day of first ovulation has been measured as early as 45 days postpartum in patients who are not breastfeeding, allowing rapid repeat pregnancy to occur [4]. And as many as 53% of patients are sexually active before 6 weeks postpartum, making family planning counseling before the postpartum visit an imperative [5]. Finally, many patients do not return for the postpartum visit. This suggests that many barriers to attending this visit exist, that the visit is not in fact necessary for women who are doing well after delivery, or indeed another mode of providing health care is necessary for patients who do not return for this visit [6, 7]. The American College of Obstetricians and Gynecologists recommends LARC be offered routinely as they have few contraindications to their use and so are safe and effective options for most women. In addition, immediate LARC placement postpartum is considered best practice [8].

The most effective implementation of a postpartum contraception program occurs within a context of shared decision making wherein patients are given full information about all options and guided to methods that are safe to use in their circumstance and best fit their lifestyle needs and desires [8]. Shared decision making also respects the family planning needs and desires of patients who do not wish to use contraception after delivery. Although the ideal time to discuss family planning has not been elucidated, analysis of the Pregnancy Risk Assessment Monitoring System determined that patients who received contraceptive counseling either prenatally or postpartum were twice as likely to choose an effective method of contraception postpartum, especially if counseling occurred both prenatally and postpartum, compared to patients who received no such counseling [9].

The informed consent process for postpartum IUD placement requires that patients understand the procedure, alternatives, benefits, and risks explained. Ideally, this should take place during prenatal visits when there is time for discussion, questions, and decision making and when the patient is not stressed by labor or other symptoms. This can be documented in the chart. When she arrives in active labor, she can reaffirm her consent and sign an informed consent form. However, patients with minimal, late, or no prenatal care may still be able to consent to IUD placement even during labor and the immediate postpartum period. Labor itself does not preclude informed consent: the patient who requires induction or augmentation of labor may have time for adequate counseling whereas the patient who arrives with strong contractions or a precipitous delivery may not. Utilizing shared decision making, regardless of the timing, will most ensure that a contraception method, if desired, is chosen without coercion or extenuating factors inappropriately influencing the patient’s decision. Contraception counseling using shared decision making significantly increases a patient’s satisfaction with her method chosen [10].

Within the scope of this article, we will focus on postpartum IUD placement and therefore will not discuss the timing and circumstances in which a variety of contraceptive options are safe to use postpartum. As a LARC method, the IUD provides reversible contraception that is highly effective for several years, safe to use by the majority of patients, easy to use, and associated with satisfaction rates significantly higher than for short-term methods [11]. IUDs are categorized in the top tier of contraceptive effectiveness by the Centers for Disease Control and Prevention (CDC), including non-hormonal and hormonal types. Contraindications to IUD use include: known or suspected cervical or intrauterine infection, known or suspected genital malignancy, or uterine cavity significantly distorted by fibroids or an anomaly [12].

The non-hormonal IUD available in the US is the copper T 380A. The release of copper ions directly in the uterus acts as spermicide to prevent pregnancy. The typical use failure rate at 1 year is 0.8%, nearly equal to its perfect use failure rate [13]. It was originally approved for use for up to 10 years, but has been proven to be effective for at least 12 years [14]. During a 3- to 6-month initial period in which the initial release of copper ions is higher, patients can experience intermenstrual bleeding and a heavier menstrual flow. Thereafter, menses are similar in timing and flow to a patient’s baseline menses. The 12-month continuation rate in adults is 85%, among the highest of all contraceptive methods [11].

There are two 52 mg hormonal IUDs available in the US. Although smaller size hormonal IUDs are also available, they have not been studied in the context of postpartum placement and will not be covered here. The release of the progestin, levonorgestrel (LNG), directly in the cervix and uterus blocks sperm from entering the cervix and inhibits sperm function within the uterus to prevent pregnancy. The typical use failure rate at 1 year is 0.2%, equal to its perfect use failure rate [13]. The brand, Mirena, is approved for use for up to 5 years, and the brand, Liletta, has been approved for use up to 4 years. Both are likely effective for at least 7 years [15]. An additional contraindication to use of the LNG IUD is recent or current breast cancer [12]. Menstrual blood loss is significantly decreased by 79 to 97% in studies [16, 17]. Patients can experience irregular menstrual bleeding, though up to 18% of patients experience amenorrhea at 1 year of use [18, 19]. Like the copper IUD, the 12-month continuation rate of the LNG IUD among adolescents and adults is among the highest of all contraceptive methods at 88% [11].

The CDC has developed the United States Medical Eligibility Criteria for Contraceptive Use (USMEC) that provides guidelines to clinicians as to when various contraceptive methods can be safely used [12]. The USMEC was adapted for the US population from the World Health Organization MEC [20]. For both MECs, recommendations are based on systematic reviews of the medical literature as well as expert opinion. Briefly, categories are assigned to each contraceptive method based on whether the benefits of use of that method outweigh the risks for certain conditions. The USMEC has assigned either category 1 (can use without restriction) or category 2 (can use as the benefits generally outweigh the risks of use) to the use of both types of IUDs in the immediate postpartum period for most patients, whether breastfeeding or non-breastfeeding. In comparison, the WHO MEC has assigned a category 3 to the use of the LNG IUD in the immediate postpartum period for breastfeeding patients globally, as the theoretical risk to breastfeeding infants may outweigh the advantages of immediate insertion in developing countries [20]. The exception to use is in the setting of puerperal sepsis (i.e. chorioamnionitis or endometritis), which is assigned a category 4 (risk is unacceptable) [12]. (Table 1) The specific evidence supporting these recommendations will be further explained. Relative contraindications for postpartum IUD placement also include postpartum hemorrhage.

**Table 1:** US medical eligibility criteria for postpartum IUD placement after vaginal or cesarean delivery

Timing of postpartum placement	Copper IUD	LNG IUD
Within 10 min of placental delivery	1	Breastfeeding = 2 Non-breastfeeding = 1
More than 10 min and less than 4 weeks	2	2
4 weeks or later	1	1
Puerperal spesis	4	4

*IUD* intrauterine device, *LNG* levonorgestrel

1 = A condition for which there is no restriction for the use of the contraceptive method

2 = A condition for which the advantages if using the method generally outweigh the advantages of using the method

3 = A condition for which the theoretical or proven risks usually outweigh the advantages of using the method

4 = A condition that represents an unacceptable health risk if the contraceptive method is used

Adapted from US Medical Eligibility Criteria for Contraceptive Use, 2016. [12]

When considering postpartum IUD placement, and the evidence for the benefits and risks of placement at various time periods, attention to defined terminology is key. Immediate postpartum placement occurs within 10 min of delivery of the placenta. Early postpartum placement is placement that occurs after 10 min and before 4 weeks after placental delivery. Interval placement is anytime 4 weeks or later after delivery [21]. The risk of IUD expulsion with immediate postpartum placement may be similar to that of early postpartum placement. Three randomized controlled trials (RCTs) that compared immediate versus early postpartum placement found no difference in expulsion rates. However, one of the trials was small (*n* = 30) and nearly all of the early placements occurred before 30 min. The other two trials resulted in conference abstracts but no full publications. There were no differences noted in: failure (i.e. unintended pregnancy), infection, uterine perforation, or other complications leading to IUD removal [22]. A systematic review of 18 studies also concluded that expulsion rates after immediate and early postpartum placement were similar, although not included were two studies from one investigator that found expulsions rates as high as 41% with early placement [23].

The risk of IUD expulsion is significantly higher with immediate postpartum placement compared to interval placement: a meta-analysis of four RCTs concluded that the risk of expulsion 6 months after IUD placement was over four times greater for immediate postpartum versus interval placement (odds ratio (OR) 4.89, 95% confidence interval (CI) 1.47–16.32). Again, there was no increased risk of: failure (i.e. unintended pregnancy), infection, or other complications leading to IUD removal [22]. The RCTs for both time comparisons included T-shaped copper and LNG IUDs, and vaginal and cesarean delivery.

Importantly, IUD usage rates are similar or slightly increased among women who received immediate postpartum IUDs compared to women who received IUDs at other times. The IUD usage rate is defined as the number of patients using an IUD at a particular point in time, even if that IUD was reinserted after a previous expulsion. Not surprisingly, there were no differences in IUD usage rates in the studies that compared immediate versus early postpartum placement [22, 24]. For the meta-analysis comparing immediate postpartum to interval placement, the IUD usage rate at 6 months was increased after immediate placement (OR 2.04, 95% CI 1.01–4.09) [22]. This may be because the increased immediate postpartum placement rate compensates for the increased expulsion rate (i.e. a significant portion of women who desire IUDs do not return for interval placement), or that women who have IUDs expelled are likely to have them replaced [22, 24]. The reasons are not fully understood and are likely multifactorial.

For breastfeeding women, postpartum IUD placement does not adversely impact breastfeeding. A systematic review found 7 RCTs that all concluded there is no decrease in breastfeeding duration or need for supplementation, and no decrease in mean infant growth or infant weight associated with the immediate postpartum placement of copper IUDs. In addition, there was no increase in expulsions associated with breastfeeding in women who received an immediate postpartum IUD [21]. Three clinical trials have investigated the use of the LNG IUD compared to the copper IUD in women postpartum and found no differences in breastfeeding or infant outcomes. Outcomes included: breastfeeding duration, need for supplementation, infant growth, and infant development. None of the studies included immediate or early postpartum placement [25]. One RCT that compared immediate postpartum versus interval placement of the LNG IUD found no difference in either breastfeeding initiation or continuation at 6 to 8 weeks postpartum. However, women who received the IUD immediately postpartum were less likely to be breastfeeding at 6 months [7].

The technique of postpartum IUD placement is sufficiently different from interval placement such that training should be provided even to clinicians who are used to placing IUDs in interval settings. At a minimum, clinicians need to be familiar with the insertion technique for each IUD type and brand they will place, as each is slightly different. Immediate postpartum IUD placement training can be provided in a group or one-on-one session, in person or via video training, and should allow models for simulated practice. The “SPIRES post partum IUD insertion training demonstration” available on youtube.com is one example of a video that provides instructions for building a postpartum uterine model and explains the technique for postpartum placement.

Both experienced clinicians and trainees can effectively place IUDs postpartum. A prospective cohort study at our institution found that a postpartum IUD program can be successfully established within a residency program at a safety net hospital. Initial training sessions were led by investigators for faculty and residents, and included both didactic lecture and hands-on training. Brief refresher training sessions were provided on the Labor and Delivery unit every 5 to 6 weeks during the study, corresponding with the beginning of each resident rotation. Ultrasound was used with all IUD placements to assist with fundal placement. The IUD expulsion rate was 17% at 6 months and did not appear to differ among faculty or residents with varied levels of clinical experience, though the study was not powered to compare outcomes as such [26]. The use of ultrasound has not yet been studied to see if it improves fundal placement or decreases expulsion rates.

Immediate postpartum placement occurs in the delivery room. Complete instructions and training materials for postpartum IUD placement are available online via the ACQUIRE project at engenderhealth.org [27]. Briefly, for vaginal delivery, once the placenta is delivered, change to a new set of sterile gloves and prep the vagina and cervix. Use a vaginal retractor or the posterior blade of a speculum to depress the posterior vagina and visualize the cervix. Place a ringed forcep on the anterior lip of the cervix. This is used to place traction on the cervix to straighten the curve of the cervical canal. The IUD is removed from its inserter and grasped gently with either a ringed or placental forcep at its vertical shaft at a slight angle away from the IUD strings. Imagine you are holding an egg: take care not to crush the shaft of the IUD. Use the forcep to place the IUD into the cervix and lower uterine segment. Drop the forcep holding the cervix and use that hand to palpate and gently depress the fundus in order to further straighten the curve of the lower uterine segment and guide the forcep/IUD to the fundus. Once at the fundus, open the forcep and move it laterally away from the shaft so as not to displace the IUD, before removing the forcep from the uterus. Cut the strings at the level of the external os. During cesarean delivery, the technique is similar, but the ringed forcep is used to place the IUD at the fundus before closure of the hysterotomy. The strings of the IUD should be tucked toward the cervical os and then the hysterotomy closed. Some clinicians use the manufacturer’s inserter rather than forceps for immediate postpartum IUD placement, although it may be more challenging to reach the fundus after vaginal delivery with the shorter copper IUD inserter.

Early postpartum placement occurs on the postpartum ward in the patient’s room or a procedure room, wherever the patient is able to lie down and place her legs in stirrups. Placement will likely be easier with a complete speculum rather than just the posterior blade. Otherwise, the technique is the same as for immediate postpartum placement. Fundal placement is more challenging as the cervix is smaller and the angle of the cervical canal more acute though uterine involution is not yet complete.

A postpartum IUD program for women who desire IUDs is cost effective over a 2-year time period postpartum [28]. To date, 18 states now provide Medicaid coverage for the IUD and its placement postpartum. Successfully implementing a postpartum IUD program requires a collaborative partnership with the hospital administration, staff, and clinicians. The Finance Department needs to know: if the state allows unbundling of the charges from the Diagnostic Related Group (DRG) for the delivery, the codes to be used specifically for postpartum placement, and how placement will be documented in the medical record. The Pharmacy will need to add the IUDs to the inpatient formulary, provide a steady supply to Labor and Delivery, and establish a process for documentation of administration. Information Services can add an order set to the electronic medical record as well as templates for electronic consent forms, procedure notes, and patient instructions. Nursing staff will be vital partners in assuring the IUD is available in the delivery room, that placement is documented, and that the patient’s questions are answered. In addition, iterative communication throughout the implementation process will be needed among team members to initiate all needed steps but also identify gaps in processes that require further attention [29].

## Conclusion

Postpartum IUD placement remains a viable option for patients who wish to use a long-acting reversible contraceptive method and to have it placed at the time of their delivery. When implemented within the context of a comprehensive and voluntary postpartum contraceptive program, postpartum IUD placement provides a highly effective method for preventing unintended pregnancy, especially for patients who may not return for the postpartum visit. The increased risk of IUD expulsion with postpartum compared to interval placement is likely countered by increased access to placement. The technique of postpartum IUD placement differs slightly from interval placement but can be easily taught. Finally, the implementation of postpartum IUD placement can be successfully accomplished in hospitals using a team-based approach and self-monitoring of outcomes.

## Abbreviations

CDC: Centers for disease control and prevention
CI: Confidence interval
DRG: Diagnostic related group
IUD: Intrauterine device
LARC: Long-acting reversible contraception
LNG: Levonorgestrel
OR: Odds ratio
RCT: Randomized controlled trial
US: United States
USMEC: United States medical eligibility criteria
WHO: World Health Organization
